# Oseltamivir and inhaled zanamivir as influenza prophylaxis in Thai health workers: a randomized, double-blind, placebo-controlled safety trial over 16 weeks

**DOI:** 10.1093/jac/dkt194

**Published:** 2013-05-08

**Authors:** T. Anekthananon, S. Pukrittayakamee, W. Ratanasuwan, P. Jittamala, P. Werarak, P. Charunwatthana, S. Suwanagool, S. Lawpoolsri, K. Stepniewska, P. Sapchookul, P. Puthavathana, C. Fukuda, N. Lindegardh, J. Tarning, N. J. White, N. Day, W. R. J. Taylor

*J Antimicrob Chemother* 2013; **68**: 697–707

The name of the second author was incorrectly spelled in the published version of this article. The correct version is shown above. The authors apologize for this error.

